# The Integrative Analysis of Thrombospondin Family Genes in Pan-Cancer Reveals that THBS2 Facilitates Gastrointestinal Cancer Metastasis

**DOI:** 10.1155/2021/4405491

**Published:** 2021-11-10

**Authors:** Chunfeng Zhang, Chenyu Hu, Kunqi Su, Kun Wang, Xiaojuan Du, Baocai Xing, Xiaofeng Liu

**Affiliations:** ^1^Department of Medical Genetics, School of Basic Medical Sciences, Peking University Health Science Center, Beijing 100191, China; ^2^Hepatopancreatobiliary Surgery Department I, Key Laboratory of Carcinogenesis and Translational Research (Ministry of Education/Beijing), Peking University Cancer Hospital & Institute, Beijing 100142, China; ^3^Department of Cell Biology, School of Basic Medical Sciences, Peking University Health Science Center, Beijing 100191, China

## Abstract

Recent cancer studies have found that the thrombospondin (THBS) family, including THBS1, THBS2, THBS3, THBS4, and THBS5, play vital roles in the development and progression of human cancers. However, their relationships with tumor stage, prognosis, and tumor immunity in pan-cancer have not been systematically reported. In the present study, we employed versatile public databases to assess the expression and mutations of different THBSs in pan-cancer and performed functional experiments to analyze the roles of THBS2 in gastrointestinal cancer metastasis. Our findings indicate that THBS genes are frequently mutated in various cancers and the dysregulation of THBS family members is associated with the progression of some cancers such as gastric cancer, colon cancer, and lung cancer. Further analyses indicate that THBS genes are associated with cancer hallmarks such as cell cycle and epithelial-mesenchymal transition (EMT). Importantly, thrombospondins, especially THBS1 and THBS2, are correlated with the immune cell infiltration level in gastrointestinal cancers. Our experiments further verified that THBS2 participates in tumor metastasis by enhancing EMT. Therefore, the overall analyses reveal that THBSs might offer us potential chances for tumor diagnosis and therapy.

## 1. Introduction

The extracellular matrix (ECM) is referred to as a complex 3D network containing a variety of macromolecules which play pivotal roles in all tissues by offering a structural platform and providing crucial communications to the cellular constituents [1]. In brief, ECM comprises collagen, glycosaminoglycans, enzymes, and glycoproteins, such as laminin and fibronectin [2]. ECM remodeling is commonly defined as a dynamic process in which cells alter the abundance, structure, and organization of individual ECM components in order to support their own activities [3]. It is well known that ECM remodeling often occurs in tumorigenesis to sustain tumor progression [4]. For example, the ECM-related proteins, MMPs (Matrix Metallopeptidase), have been identified as prognostic indicators in lots of cancers since the dysfunction of MMPs facilitates cancer cell survival, metastasis, and angiogenesis [5–7]. Thus, the understanding of the roles of ECM-related proteins in cancer biology will be helpful for providing new approaches in the improvement of cancer therapeutics [8].

The thrombospondin (THBS) family comprises a group of ECM proteins, including THBS1, THBS2, THBS3, THBS4, and THBS5 [9]. Based on their molecular structure, the five THBSs were divided into two groups [10]. The first group includes thrombospondin 1 (THBS1) and thrombospondin 2 (THBS2). The other group consists of three pentameric proteins, thrombospondin 3 (THBS3), thrombospondin 4 (THBS4), and cartilage oligomeric matrix protein (COMP, also called THBS5). A series of studies have demonstrated that THBSs participate in multiple biological processes including tissue remodeling, angiogenesis, and neoplasia [11–13]. In tumorigenesis, the roles of THBSs are multifaceted, and the underlying mechanisms are sophisticated. At the early stage of carcinogenesis, THBS1 and THBS2 secreted by normal tissue act as an antiangiogenic wall [14, 15]. However, with tumor progression, they may switch to enhance angiogenic phenotype in order to support tumor development and metastasis [16, 17]. High expression of THBS3 maintains the capacity of angiogenesis and promotes tumor progression in osteosarcoma [18]. THBS4 is upregulated in colorectal cancer and is associated with tumor progression [19, 20]. Moreover, THBS4 participates in HCC progression by FAK/PI3K/AKT pathway [21]. THBS5 expression is significantly higher in papillary thyroid carcinoma tissues and is associated with tumor cell migration and invasiveness [22]. However, the dysregulated expression of the THBS family and their relationships with tumor prognosis and tumor immunity in pan-cancer have not been comprehensively reported.

In the present study, we carried out this study using the data from The Cancer Genome Atlas (TCGA) and other public databases to evaluate the mutations and expression levels of different THBSs in pan-cancer, along with their functional networks, prognostic values, and tumor-immune interactions. Our study aimed to uncover the latent diagnostic, prognostic, and therapeutic values of THBSs in pan-cancer. The results in different databases were validated with each other to make the results more convincing. Finally, we also selected THBS2 as a representative to study its potential roles in cancer metastasis and EMT by functional experiments.

## 2. Materials and Methods

### 2.1. Cell Culture and Transfection

Cell lines (LOVO, BGC-823) were obtained from the Cell Resource Center (National Infrastructure of Cell Line Resource, NSTI) and cultured in a DMEM medium. All medium was supplemented with 10% fetal bovine serum. Cells were maintained in 5% CO_2_ at 37°C in incubators with 100% humidity. Cells were transfected with siRNA RNA duplexes by Lipofectamine 2000 (Invitrogen) according to the manufacturer's protocol. For silencing THBS2 expression, siRNA (small interference RNA) targeting THBS2: 5′-CAGCACCACUGCUGAGAAUAAGA-3′ was chemically synthesized together with an unrelated control siRNA (5′-ACUACCGUUGUUAUAGGUG-3′) (Shanghai GenePharma Co., Ltd).

### 2.2. Total RNA Extraction and Real-Time Quantitative PCR

Total RNA was extracted from cells using TRIzol reagent (Invitrogen) as previously described [23]. Then, the cDNA library was synthesized, and qPCR was performed according to Minimal Information for Publication of Quantitative Real-Time PCR Experiments (MIQE) guidelines.

### 2.3. Western Blot

Western blot was performed as previously described [24]. The anti-THBS2 polyclonal antibody was purchased from BOSTER (BA3616-2, China). Anti-*β*-actin antibody was purchased from Abclonal Technology (AC026, China).

### 2.4. Transwell Migration Assay

Cell migration assay was conducted in 24-well transwell chambers with 8 *μ*m pore polycarbonate filter inserts (Corning Inc., USA). Briefly, cells were counted and seeded in uncoated inserts. The lower chambers were added with 10% FBS-supplemented DMEM as a chemoattractant. Finally, cells on the upper side of the filter were discharged and those on the lower surface of the insert were fixed in 4% PFA (paraformaldehyde) and stained with crystal violet (Beyotime Biotechnology, China). Then, the migrated cells were counted and summarized.

### 2.5. Wound-Healing Assay

Cells were transfected as indicated and seeded into 6-well plates. When reaching 90% cell confluence, a straight line was drawn on the cell monolayer. After washing the cell debris with PBS, cells were incubated with serum-free DMEM at 37°C. Images of migratory cells were captured at indicated time points under a light microscope. The relative cell migration rate was analyzed using ImageJ software.

### 2.6. Gene Expression Profiling Interactive Analysis

GEPIA (https://gepia.cancer-pku.cn/) is a gene expression analysis tool which includes 9 736 tumors and 8 587 normal samples from the TCGA and the GTEx databases [25]. Here, we employed GEPIA to compare the expression levels of the thrombospondin family in 31 kinds of TCGA cancers.

### 2.7. Survival Analysis

We used GEPIA to perform the overall survival (OS) and disease-free survival (DFS) analyses. The log-rank test was performed for computing *p* value, and *p* < 0.05 was regarded as significant.

### 2.8. cBio Cancer Genomics Portal (cBioPortal) Analysis

cBioPortal (https://www.cbioportal.org/) converts molecular information of cancer tissues and cell lines into genetic, epigenetic, and gene expression data [26]. We employed it to analyze the THBS mutations in pan-cancer.

### 2.9. Methylation and Clinical Analyses

The correlation between methylation of THBS genes and the THBS expression levels was determined for TCGA cancers using data obtained from GSCALite (https://bioinfo.life.hust.edu.cn/web/GSCALite/) [27].

### 2.10. Transcriptional Factors and Epigenetics Analyses

We downloaded data for transcriptional factors and chromatin remodeling factors with binding sites positioned within 1 kb upstream or downstream of THBS family members from ChIPBase V2.0 (https://rna.sysu.edu.cn/chipbase/) [28]. The coexpression between crucial factors and THBS2 was evaluated in human cancers. miRNA-targeted gene data was downloaded from miRWalk (https://mirwalk.umm.uni-heidelberg.de/) [29]. The miRNAs bound to the THBS family were acquired from STARBASE v3.0 (https://starbase.sysu.edu.cn/) [30]. In STARBASE v3.0, the correlation between the expression of miRNAs and the expression of THBS2 in human cancers was investigated.

### 2.11. LinkedOmics Database Analysis

LinkedOmics (https://www.linkedomics.org/) contains multiomics and clinical data for 32 types of cancers from TCGA [31]. We employed LinkedOmics to analyze the relationships between clinical characteristics and the expression level of THBS family members.

### 2.12. Drug and Pathway Analyses

The global percentage maps of the THBS genes in cancer-related pathways were obtained from GSCALite (https://bioinfo.life.hust.edu.cn/web/GSCALite/). All data for THBS family-related drugs were obtained from PharmacoDB (https://pharmacodb.pmgenomics.ca/) [32].

### 2.13. Tumor Immunology Analysis

Tumor Immune Estimation Resource (TIMER, https://cistrome.shinyapps.io/timer/) was used to explore immune infiltrates in a diversity of cancers systematically [33]. We utilized it to analyze the relations between gene expression and immune infiltration.

### 2.14. PPI Network and Hub-Gene Analyses

The protein-protein interactions network was constructed by STRING v11 (https://string-db.org/), which is an online database and contains comprehensive information of multiple proteins [34]. Cytoscape (https://www.cytoscape.org/) version (3.7.1) [35] was used to visualize the PPI networks. The hub genes were selected using cytoHubba plug-in. A confidence score ≥0.4 was selected as the cut-off criteria.

## 3. Results

### 3.1. Mutation Analysis of Thrombospondin Family in Pan-Cancer

To analyze the mutation of the thrombospondin family in pan-cancer, we assessed the mutation information in pan-cancer from the cBioPortal (http://www.cbioportal.org/). Using the TCGA PanCancerAtlas for Mutual Exclusivity analysis of pan-cancer mutations, we uncovered cooccurrence relationships of THBS2, THBS4, and THBS5 with THBS1; THBS4, THBS3, and THBS5 with THBS2; and THBS3 and THBS4 with THBS5. All relationships showed statistical significance (*p* < 0.05), but no cooccurrence relationship was found in any TCGA single cancer.

**Figure 1:**
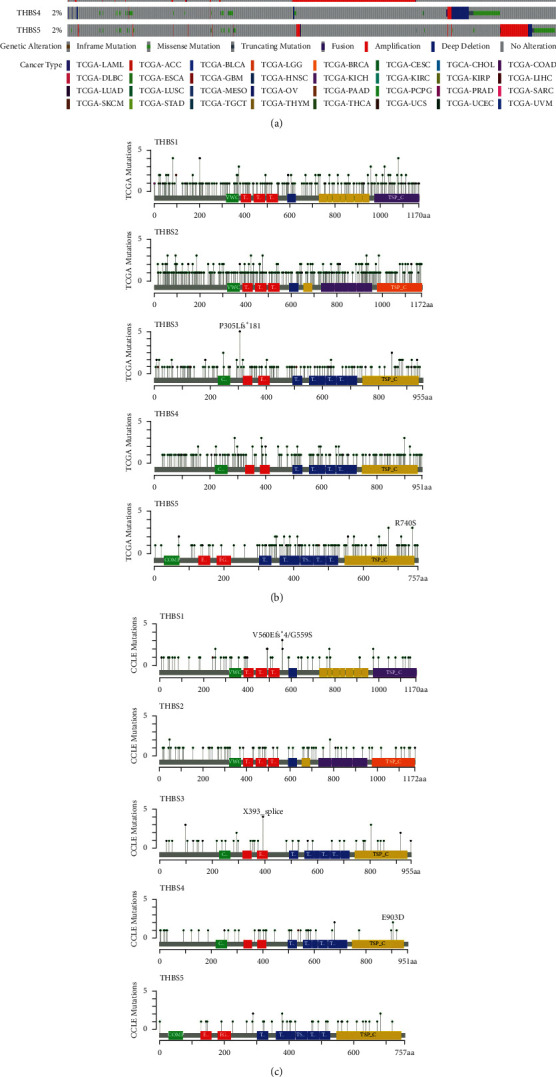
The mutations of the thrombospondin family in cancer. (a) THBSs exhibit nonsynonymous mutations in the coding region in TCGA pan-cancer. Each gray vertical bar represents a patient. (b) The mutation distribution of THBSs in TCGA cancer. (c) Amino acid mutation of THBSs in Cancer Cell Line Encyclopedia (CCLE).

At the cancer level, mutations of thrombospondins were sporadic and were discovered in the 33 cancers included in TCGA. The mutation occurrence of THBS1, THBS2, THBS3, THBS4, and THBS5 was 2.7%, 4%, 5%, 2%, and 2%, respectively (Figure 1(a)). Melanoma displayed the highest number of thrombospondin mutations, followed by uterine carcinosarcoma, cholangiocarcinoma, and liver cancer. The total mutation rates of thrombospondin members in the above five cancers were 27.93%, 26.65%, 22.81%, 22.22%, and 20.7%, respectively. Hot spots of mutations in the different genes were further analyzed as shown in Figure 1(b). The mutations in THBS1, THBS2, THBS3, THBS4, and THBS5 were mainly in the thrombospondin type 1, thrombospondin type 3, and TSP_C domain. The majority of spot mutations were predicted to be missense for the functional and structural importance of these proteins. However, the hotspot mutation G203Afs*∗*13 of THBS1 was found in three patients with STAD (stomach adenocarcinoma), which encodes a truncated form. P305Lfs*∗*181, one of the hotspot mutations of THBS3, was detected in six patients with STAD or UCEC (uterine corpus endometrial carcinoma), and this change was damaging. The hot spot mutation X399_splice of THBS4 occurred in two patients with SKCM (skin cutaneous melanoma) and also encodes a truncated protein. The mutation distribution of THBS family members of CCLE showed that multiple mutations were also found in TCGA cancers and the majority of mutations were concentrated in the TSP-type domain (Figure 1(c)).

### 3.2. Expression and Clinical Analyses of Thrombospondins in Pan-Cancer

**Figure 2:**
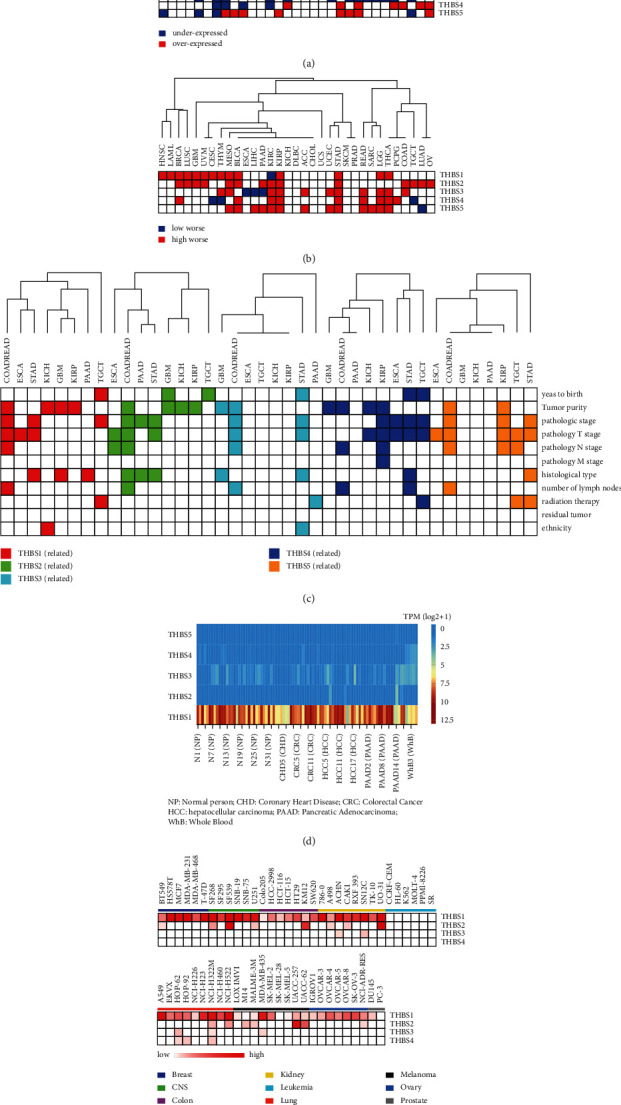
THBSs' expression and clinical features. (a) Differential expression of THBSs between TCGA cancer and the normal tissues. Blue represents low expression in cancer tissue (*p* < 0.05), and red indicates high expression in cancer tissue (*p* < 0.05). (b) Overall survival analysis between high and low expression of THBSs in TCGA cancers. The blue indicates that the survival of patients with THBSs low expression in cancer tissue is shorter (*p* < 0.05). The red indicates that the survival of patients with THBS high expressions in cancer tissue is worse (*p* < 0.05). (c) The relationship between THBS expression levels and the clinical parameters in TCGA cancers. The color indicates that clinical parameters were significantly associated with gene expression level (*p* < 0.05), and the white means the opposite. (d) The heatmap of THBS expressions in blood derived from the indicated patients. (e) The heatmap of THBS1, THBS2, THBS3, and THBS4 as extracellular vesicle proteins detected in 60 cell lines of NCI-60.

Differential expression analysis of 31 kinds of TCGA cancers was performed by GEPIA. As shown in Figure 2(a), we presented significant differential expression between adjacent noncancerous tissue and cancer tissue (*p* < 0.05). THBS1 was upregulated in ESCA (esophageal carcinoma), GBM (glioblastoma multiforme), and PAAD (pancreatic adenocarcinoma) and downregulated in 13 types of cancers, including LUSC (lung squamous cell carcinoma) and KICH (kidney chromophobe). THBS2, THBS3, THBS4, and THBS5 were differentially expressed in TGCT (testicular germ cell tumors) and COAD (colon adenocarcinoma). THBS3 was mainly downregulated in 13 types of cancers, including COAD, LUSC, and LUAD (lung adenocarcinoma).

Next, we assessed the relations of THBS expressions with overall survival in TCGA pan-cancer by Kaplan-Meier Plotter and GEPIA. As shown in Figure 2(b), high expression of THBSs mostly predicated worse survival of cancers. In pan-kidney cancers (KIRP and KIRC) and gastric cancer, all members of the THBSs family were associated with overall survival. The high expression of THBS2 was correlated with worse survival in fourteen types of cancers. Combined with the expression analysis, we found that the expression level of the THBS family was consistent with its relation to survival in nine types of cancers. THBS2 was upregulated in COAD, GBM, KIRC, KIRP (kidney renal papillary cell carcinoma), PAAD, STAD, and TGCT, and overexpression of THBS2 was associated with shorter survival in these cancers. THBS1 was upregulated in GBM, and high THBS1 expression was correlated with the poor prognosis of GBM patients. THBS3 was downregulated in ESCA, and low expression predicated worse survival in ESCA. THBS5 was upregulated in PAAD, STAD, and READ, and high expression of THBS5 was associated with worse survival in these cancers.

We then performed a comprehensive expression-clinical analysis of all members of the THBS family in these cancers through LinkedOmics (Figure 2(c)). In colorectal cancer, all of THBSs were related to tumor purity and pathological stage. The expression of THBS1, THBS2, THBS3, THBS4, and THBS5 was related to pathology T stage in STAD, and that of THBS1, THBS3, and THBS5 was related to radiation therapy in TGCT, PAAD, and STAD, respectively.

The expression data of THBS family members in blood from the normal person (NP) or patients with coronary heart disease (CHD), colorectal cancer (CRC), hepatocellular carcinoma (HCC), or pancreatic cancer (PAAD) was downloaded from exoRBase (Figure 2(d)). THBS2, THBS4, and THBS5 were lowly expressed in blood. Conversely, the expression of THBS1 and THBS3 was relatively high in the blood. Moreover, the expression of THBS1 was highly expressed in the blood of CRC and PAAD. By assessing the data for 60 cell lines at the National Cancer Institute (NCI-60) (Figure 2(e)), we found that THBS1 protein is present in extracellular vesicles derived from the breast, CNS (Central Nervous System), colon, kidney, lung, melanoma, and ovarian cancer cells. THBS2 protein appears in extracellular vesicles from CNS, colon, kidney, and melanoma cancer cells.

### 3.3. Methylation, Drug, and Pathway Analyses of THBS Family in Pan-Cancer

**Figure 3:**
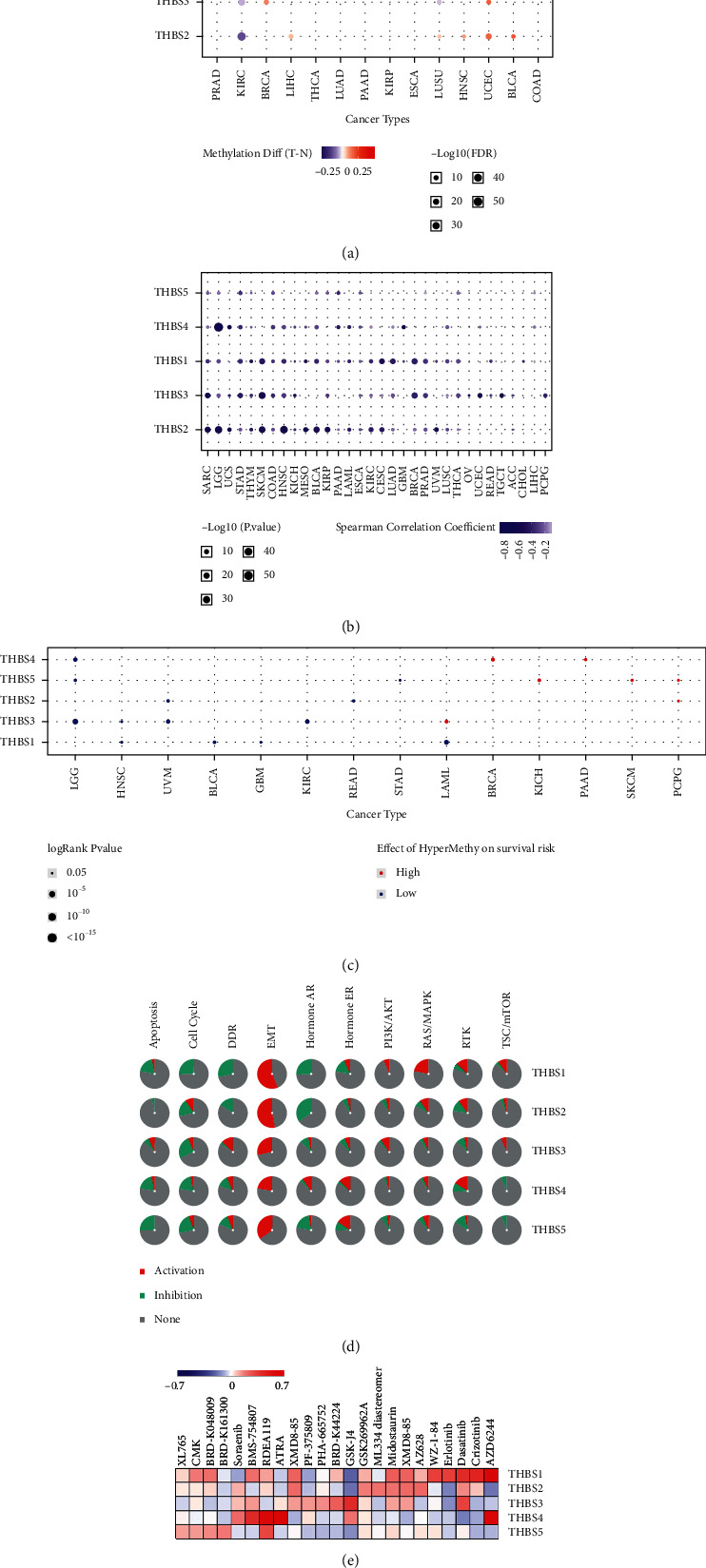
THBSs' methylation and distribution in cancer samples. (a) Bubble map of the differential methylation of THBS genes between TCGA cancer and normal samples. Blue dots indicate methylation downregulation in tumors, and red dots represent methylation upregulation in tumors. (b) The correlation between methylation level and THBS expression levels in TCGA cancer. (c) The relations of high or low methylation of THBS genes with overall survival in TCGA cancers. Only relations with *p* value < 0.05 are displayed on the diagram. Red dots indicate that hypermethylation predicts worse survival in tumors, while blue dots represent the opposite. (d) Global percentage map shows the percentage of the gene's function (activation or inhibition) for each pathway in all cancers. (e) Heatmap displaying the predicted drug sensitivity of cancers with upregulated THBSs expression.

We analyzed the methylation of the THBS genes in TCGA cancers using GSCALite. Here, we found downregulated methylation of THBS1 in BRCA, THBS2 in KIRC, and THBS4 in PRAD (prostate adenocarcinoma) and LIHC (liver hepatocellular carcinoma) (Figure 3(a)). We observed upregulated methylation of THBS1, THBS4, and THBS5 in COAD. Additionally, the expression of the THBS family was mainly negatively correlated with methylation (Figure 3(b)). The relationship between methylation and survival showed hypomethylation of THBS1 predicted worse survival in GBM and hypomethylation of THBS5 indicated poorer survival in STAD, which is consistent with the correlation of high expression of THBS1 in GBM or upregulation of THBS5 in STAD with worse survival of the patients (Figure 3(c)). These results indicate that the carcinogenic mechanism of THBS1 in GBM and THBS5 in STAD might be related to promoter methylation.

We next analyzed the THBSs-related pathways in human cancers by GSCALite (Figure 3(d)). THBS1 exhibited the strong activation of EMT and RAS/MAPK response and showed a complete inhibitory effect on the cell cycle and DDR (DNA damage repair). THBS2 displayed the strong activation of EMT and the strong inhibition of hormones AR. In general, THBS3, THBS4, and THBS5 also showed strong activation of EMT.

The list of THBS family-related cancer drugs was gained from the cancer pharmacogenomics research database, PharmacoDB, using *p* < 0.05 and standardized coefficient >0.1 as cut-off values. The five most sensitive cancer drugs for each THBS were shown in Figure 3(e). High expression of THBS1 or THBS4 is more sensitive to AZD6244, the inhibitor of MEK1 used to treat NSLC (nonsmall-cell lung cancer). High expression of THBS1 or THBS3 tends to be more sensitive to Dasatinib. Dasatinib is a novel multitarget inhibitor to treat CML (chronic myelogenous leukemia) and ALL (acute lymphatic leukemia). High expression of THBS2 is sensitive to AZ628, an inhibitor of pan-Raf. These results suggest that rational use of inhibitors might be more effective in treating tumors with high expression of certain THBS family members.

### 3.4. Upregulation of THBS2 mRNA Expression Is Associated with Progression of Gastric, Colon, and Pancreatic Cancer

**Figure 4:**
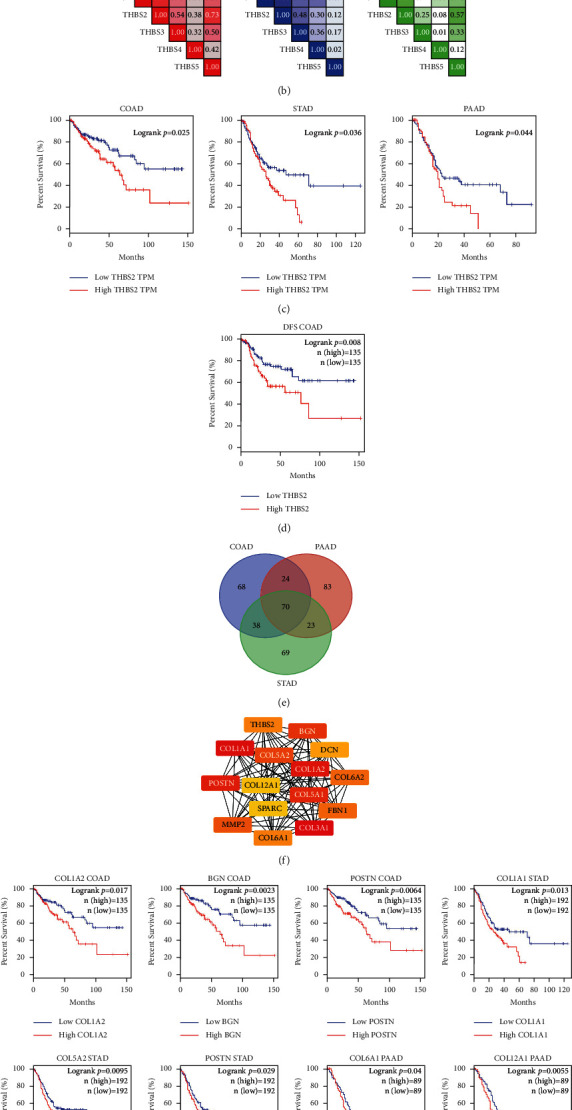
Upregulation of THBS2 mRNA expression is associated with the progression of gastric, colon, and pancreatic cancer. (a) The expression level of THBS2 in COAD, STAD, PAAD, and corresponding normal tissues. (b) The relationships of THBS2 with THBS1, THBS3, THBS4, and THBS5 in COAD, STAD, and PAAD. (c) Overall survival curve of THBS2 in the colon, gastric, and pancreatic cancer. (d) Disease-free survival curve of THBS2 in COAD. (e) Venn diagram displaying the overlapped genes among THBS2-coexpressed genes in COAD, STAD, and PAAD. (f) Visualization of protein-protein interaction (PPI) networks for the top 15 hub overlapped genes from (e). (g) The relationships of hub genes (*COL1A2*, *BGN*, *POSTN*, *COL1A1*, *COL5A2*, *COL6A1*, and *COL12A1*) and overall survival in STAD, COAD, or PAAD.

Next, THBS2 was selected as a representative for further study. We analyzed the roles of THBS2 in the gastric, colon, and pancreatic cancer, since THBS2 is upregulated in these three cancers (Figure 4(a)). Correlation analyses using TCGA data showed that THBS2 is significantly positively correlated with THBS1, THBS3, THBS4, and THBS5 in colon cancer. In gastric cancer, THBS2 is positively correlated with THBS1, THBS3, and THBS4. In pancreatic cancer, THBS2 is correlated with THBS1, THBS3, and THBS5 (Figure 4(b)). These data suggested that the THBS family might function in tumorigenesis together. The analysis of the optimal survival curve revealed that high expression of THBS2 predicts worse overall survival in COAD, STAD, and PAAD (Figure 4(c)). Moreover, the overexpression of THBS2 is associated with shorter disease-free survival (DFS) in colon cancer (Figure 4(d)).

To further analyze how THBS2 functions in tumorigenesis, we obtained the top 200 THBS2-coexpressed genes for COAD, STAD, and PAAD through GEPIA. The coexpressed genes obtained from these three cancers were cross-referenced to obtain the common coexpressed genes (Figure 4(e)). We identified the top 15 hub genes among the common genes (Figure 4(f)). These hub genes play pivotal roles in cell adhesion and migration, consistent with the pathway analysis that THBS2 had the strong activation of EMT. We also performed the survival analyses of these hub genes using TCGA datasets (Figure 4(g)). High expression of *COL1A2*, *BGN*, or *POSTN* was associated with worse survival in COAD. Upregulation of *COL1A1*, *COL5A2*, or *POSTN* was correlated with shorter survival in STAD. Overexpression of *COL6A1* or *COL12A1* predicted poorer survival in PAAD. These data indicated that THBS2 might work with different partners in different types of cancer.

### 3.5. THBS2 Shows the Ability to Promote EMT and Tumor Metastasis

**Figure 5:**
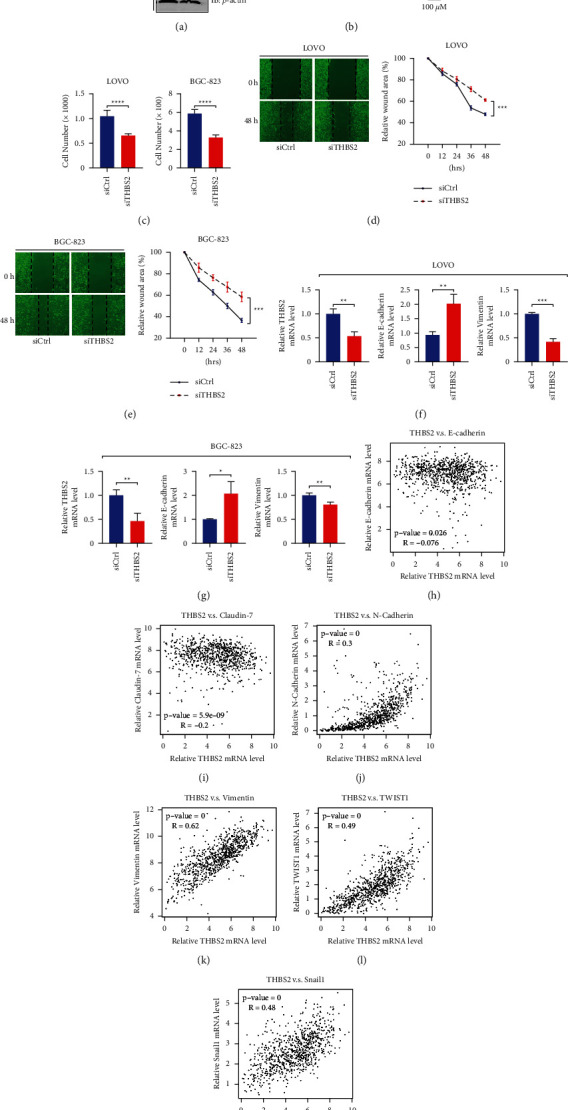
THBS2 promotes cancer cell EMT and metastasis. (a) LOVO or BGC-823 cells were transfected with the indicated siRNAs. Then, a western blot was performed to evaluate the THBS2 protein level. (b) The cells were transfected with indicated siRNAs. Then, a transwell migration assay was carried out to assess cell migration. (c) The transwell migration assay in (b) was summarized (^*∗∗∗∗*^*p* < 0.0001). (d-e) Cells were transfected with the indicated siRNAs. A wound-healing assay was performed to analyze cancer cell migration (^*∗∗∗*^*p* < 0.001). (f) LOVO cells were transfected with indicated siRNAs. Then, total RNAs were extracted, and RT-qPCR was performed for the indicated genes (^*∗∗*^*p* < 0.01). (g) BGC-823 cells were transfected with indicated siRNAs. Then, total RNAs were extracted, and RT-qPCR was performed for the indicated genes (^*∗*^*p* < 0.05, ^*∗∗*^*p* < 0.01). (h–m) The correlation between THBS2 expression and the expression level of critical EMT marker genes in colon, gastric, and pancreatic cancers.

To further verify whether THBS2 regulates tumor metastasis, we performed more experiments using colorectal and gastric cancer cells. As shown in Figure 5(a), we depleted THBS2 with siRNAs in the human colorectal cancer cell, LOVO, and gastric cancer cell, BGC-823. Importantly, depletion of THBS2 significantly decreased the migration of cancer cells (Figures 5(b) and 5(c)). Consistently, wound-healing assay also suggested that knockdown of THBS2 attenuated cancer cell migration (Figures 5(d) and 5(e)). These data suggested that THBS2 has the ability to promote cancer cell migration. We further examined the expression of EMT markers after THBS2 depletion. As shown in Figures 5(f) and 5(g), the knockdown of THBS2 upregulated the expression of the epithelial marker, E-cadherin, and downregulated the expression of a mesenchymal marker, Vimentin. In addition, correlation analyses using TCGA-STAD, COAD, and PAAD cohort suggested that THBS2 was negatively correlated with E-cadherin or Claudin-7 (the markers of epithelial cells) (Figures 5(h) and 5(i)), while THBS2 was positively correlated with the expression level of N-cadherin, Vimentin, TWIST1, or Snail1 (the mesenchymal markers) (Figures 5(j)–5(m)). Thus, these results reveal that THBS2 could promote tumor metastasis by enhancing EMT, consistent with the analysis from GSCALite.

### 3.6. Transcription and Epigenetics Analyses of THBS Genes in Pan-Cancer

**Figure 6:**
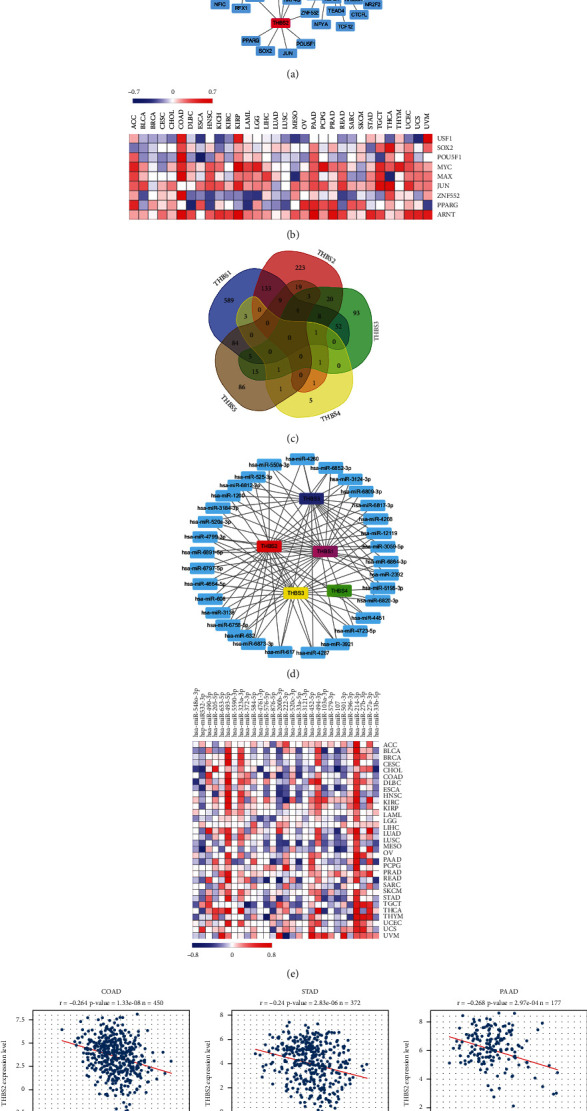
Transcription and epigenetics analyses of THBS genes in pan-cancer. (a) The network of THBS genes and their related TFs. (b) The expression correlation of THBS2 and its typical TFs in pan-cancer. (c) Venn diagram showing THBS genes-related miRNAs. (d) The network of THBS genes and the miRNAs targeting at least three THBSs. (e) The expression correlation heatmap between 29 miRNAs and THBS2 in pan-cancer. (f) The correlation analysis of THBS2 expression with hsa-miR-532-3p expression level in COAD, STAD, and PAAD.

Next, we analyzed transcriptional modulation of thrombospondin family genes in pan-cancer. We obtained the transcriptional factors (TFs) and chromatin remodeling factors with binding sites located within the 1 kb upstream or downstream of the promoter of each THBS family member from ChIPBase V2.0 (Figure 6(a)). Oncogenes, like *MYC* and *MAX*, are involved in regulating the transcription of THBS1, THBS2, THBS3, or THBS4. The correlation of TFs expression and THBS2 expression was assessed in pan-cancer as shown in Figure 6(b). MYC, SOX2, JUN, or ARNT was significantly correlated with THBS2 in most cancers, including COAD and PAAD. Previous studies have demonstrated that these transcriptional factors play important roles in facilitating cancer progression [36]. These results indicate that THBS2 may be transcriptionally activated in these cancers.

Using miRWalk, we predicted a total of 1356 miRNAs that potentially target the THBS transcripts (Figure 6(c)). In terms of the total number of miRNAs targeting a single gene, THBS1 could be targeted by the highest number of miRNAs, followed by THBS2 and THBS5. THBS family members mostly bound to a specific miRNA, but 26.5% of miRNAs bind to multiple thrombospondins. Statistical analysis indicated that 32 miRNAs were associated with at least three members of the thrombospondin family (Figure 6(d)). Comparing these miRNAs with the data from STARBASE v3.047, 29 miRNA were included in both databases. We further analyzed the expression correlation between these 29 miRNAs and THBS2 in pan-cancer (Figure 6(e)). We found that half of the miRNAs exhibit an inhibitory effect on THBS2 in ESCA and LUSC. Moreover, hsa-miR-532-3p had a consistent inhibitory effect on THBS2 in COAD (*r* = −0.264), STAD (*r* = −0.24), and PAAD (*r* = −0.268) (Figure 6(f)).

### 3.7. THBS2 Expression Is Correlated with Immune Infiltration Level in Colon, Gastric, and Pancreatic Cancers

**Figure 7:**
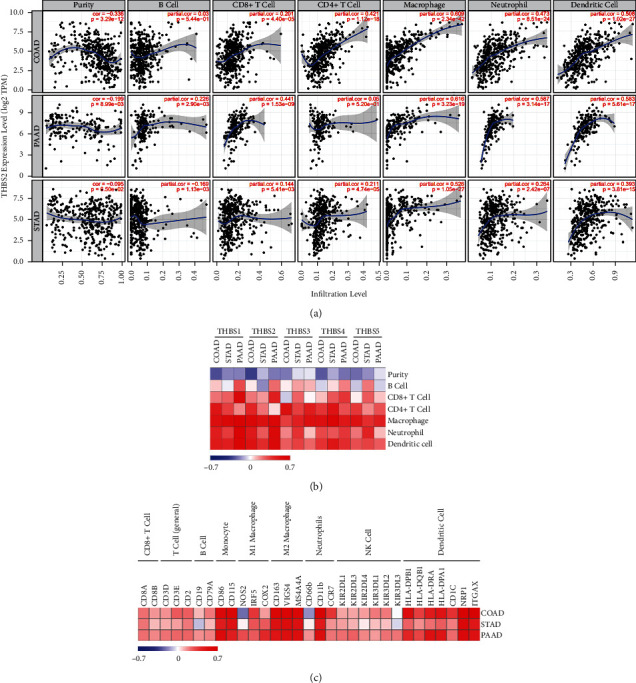
The correlation between THBSs and immune infiltration level in cancers. (a) The correlation analysis between the expression level of THBS2 and immune cells in COAD, STAD, and PAAD using the TIMER data portal. (b) The correlation heatmap between immune infiltration level and THBSs in cancer. (c) The correlation heatmap between THBS2 expression and the expression of immune cell markers.

Since EMT is associated with tumor-infiltrating lymphocytes [37], one of the independent predictors of sentinel lymph node status and survival in cancers, we next investigated the relationship between THBS2 and the diverse immune infiltrating cells using TIMER. As shown in Figure 7(a), the THBS2 expression level was negatively correlated with tumor purity in COAD, STAD, and PAAD. THBS2 expression level has significant positive correlations with infiltrating levels of CD8^+^ T cells, CD4^+^ T cells, macrophages, neutrophils, and DCs (dendritic cells) in COAD. Moreover, the THBS2 expression level was significantly associated with the infiltrating levels of B cells, CD8^+^ T cells, macrophages, neutrophils, and DCs in PAAD. Similar results were also observed in STAD. These findings suggest that THBS2 may play a specific role in immune infiltration, especially in the infiltration of CD8^+^ T cells, macrophages, neutrophils, and DCs, in colon, pancreatic, and gastric cancers.

We also analyzed the relations of the thrombospondin family with immune cell infiltrations in COAD, STAD, and PAAD. As shown in Figure 7(b), all thrombospondins were negatively correlated with tumor purity. Moreover, all of these thrombospondins, especially THBS1 and THBS2, had positive correlations with infiltrating levels of macrophages, neutrophils, and DCs. These data further indicate that thrombospondins are involved in immune infiltration in cancer. We further focused on the correlations between THBS2 and immune marker sets of various immune cells in COAD, STAD, and PAAD. Importantly, we found that the expression levels of most marker sets of monocytes, M2 macrophages, neutrophils, and DCs have strong correlations with THBS2 expression (Figure 7(c)). This is consistent with the results that THBS2 expression is strongly related to infiltration levels of macrophages, neutrophils, and DCs. Tumor-associated macrophages (TAMs) and DCs have been found to promote metastasis [38]. Thus, it is possible that THBS2 is an important factor mediating tumor-associated macrophages/DCs and tumor metastasis.

## 4. Discussion

In this study, we analyzed the dysregulated expression of the THBS family and their relations with tumor stage, prognosis, and tumor immunity in pan-cancer using versatile public databases. We mainly found that THBS1, THBS2, and THBS3 are dysregulated in various cancers, and their expression level is associated with the prognosis of patients. Furthermore, pathway analyses revealed that THBS members display the potential to activate EMT in cancers. Particularly, THBS1 and THBS2 have an active effect on the EMT pathway in more than half of cancer types. Previous studies have reported that THBS1 promotes tumor metastasis by enhancing EMT [11, 39, 40]. Thus, we chose THBS2 as a representative to further explore the roles of THBS2 in EMT and tumor metastasis by cellular experiments. We found that inhibition of THBS2 significantly attenuated cancer cell EMT and decreased cancer cell migration. Additionally, upregulation of THBS2 mRNA expression is associated with the progression of gastric, colon, and pancreatic cancer. These results revealed that THBS2 is involved in gastrointestinal cancer metastasis by enhancing EMT. It needs to be further explored whether other THBS genes, including THBS3, THBS4, and THBS5, participate in modulating cancer cell EMT and metastasis in the future study. Finally, all the THBS members are correlated with dominant immune cells' infiltration more or less.

THBS1 plays a complex role in tumor development and progression. THBS1 is overexpressed in the invasive phenotypes of melanoma and invasive ductal breast cancer [39, 41]. In oral squamous cell carcinoma cells, THBS1 upregulated the expression of MMPs and promoted cancer cell migration and invasion [40]. Increased THBS1 is associated with colorectal cancer liver metastasis and THBS1 promotes colorectal cancer cell metastasis by enhancing EMT [11]. Moreover, specific inhibition of the THBS1/CD47 interaction by an antagonist peptide attenuates cell invasion glioblastoma [42]. Consistently, we also found that high expression of THBS1 is associated with the progression of many cancers, including HNSC (head and neck squamous cell carcinoma), BRCA (breast invasive carcinoma), and GBM. Mechanistically, we uncovered that THBS1 exhibited the strong activation of EMT and RAS/MAPK response, along with showing an inhibitory effect on the cell cycle and DDR. Moreover, the expression of THBS1 is tightly associated with the infiltration of immune cells. Combined with the previous studies, our results uncovered the prognostic, diagnostic, and therapeutic values of THBS1 in human cancers.

THBS2 is a multifunctional glycoprotein involved in various cellular processes. Several studies have reported that the mRNA levels of THBS2 were elevated in tumor tissues and that higher THBS2 expression was associated with a worse prognosis of patients [43, 44]. Consistently, we also found that THBS2 is upregulated in colon cancer, gastric cancer, and pancreatic cancer using the TCGA database. Overexpression of THBS2 predicts short overall survival in these three cancers. THBS2 shows the potential ability to enhance EMT, which is critical in tumor metastasis, indicating that THBS2 may be involved in tumor metastasis. In urothelial carcinomas, THBS2 high expression was significantly associated with nodal metastasis and vascular invasion [44]. In colorectal cancer, high THBS2 expression was significantly correlated with lymph node metastasis [43]. Together with these studies, our results identified THBS2 as a potential stimulator in human colorectal, gastric, and pancreatic cancer metastasis. We further verified our results with functional experiments. Depletion of THBS2 significantly inhibits cancer cell migration and attenuates EMT phenotypes. Correlation analyses suggested that THBS2 expression is significantly correlated with the expression of EMT markers. Moreover, THBS2 is also correlated with tumor-associated immune cell infiltrations. These results suggest that THBS2 might participate in the progression of STAD, COAD, and PAAD. In the future study, it will be more convincing and meaningful to demonstrate the role of THBS2 in tumor metastasis through knockout or knock-in animal models.

To date, the research about the roles of THBS3 in cancer is poor although THBS3 has been reported to be associated with the prognosis of patients with osteosarcoma and gastric cancer [18, 45]. Using pan-cancer data, we found that THBS3 is downregulated in 13 kinds of cancers while overexpression of THBS3 predicts poor outcomes in 11 kinds of cancer. Interestingly, THBS3 is also be predicted to participate in the activation of EMT and immune cell infiltrations in human cancers. Thus, the roles of THBS3 need to be further determined in future studies.

THBS4 is an important extracellular secreted glycoprotein modulating the organization, repair, and remodeling of ECM [46]. It is reported that THBS4 has a strong correlation with histological type, since it is extensively overexpressed in the diffuse type and generally lacks intestinal type [47]. THBS4 mRNA and protein levels were upregulated in human gastric cancer cells compared to normal gastric cells, and THBS4 overexpression promoted the migration and invasion of gastric cancer cells [48]. In HCC, THBS4 promotes cancer progression via FAK/PI3K/AKT pathway [21]. In colorectal cancer, THBS4 is critical in PDGFR*β*-mediated tumor progression [19]. Here, we found that THBS4 is upregulated in gastric cancer and overexpression of THBS4 predicts shorter overall survival of gastric cancer patients. The research underlying the mechanism of THBS4 in tumorigenesis is rare. TGF-*β*1 increases the THBS4 expression and affects angiogenesis to promote tumor growth in endothelial cells [49]. Our data suggest that THBS4 might be involved in regulating cancer cell EMT phenotypes.

THBS5 acts as a soluble glycoprotein expressed in cartilage. THBS5 expression is significantly higher in papillary thyroid carcinoma tissues and promotes migration and invasiveness of papillary thyroid carcinoma cells [22]. Bioinformatics studies revealed that THBS5 was a potential prognostic marker for gastric cancer [15, 45]. These studies are similar to our results. We found that THBS5 is upregulated in 8 kinds of cancers and overexpression of THBS5 is associated with prognosis in 13 types of cancers. Moreover, THBS5 might be involved in cancer cell EMT. More studies on the roles of THBS5 are required to understand its significance to cancer therapy.

By integrating multiple databases, our study indicates that THBS family members are associated with the progression of many cancers such as gastric cancer, colon cancer, and lung cancer. Moreover, thrombospondins, especially THBS1 and THBS2, are associated with immune cell infiltration levels in gastrointestinal cancers. Our experiments further suggest that THBS2 is involved in tumor metastasis by enhancing EMT. These findings may deepen our understanding of the potential roles of THBS family members in cancer progression.

## Data Availability

The datasets used and/or analyzed during the current study are available from the corresponding author on reasonable request.

## References

[1] Garde A., Sherwood D. R. (2021). Fueling cell invasion through extracellular matrix. *Trends in Cell Biology*.

[2] Cruz-Acuña R., Vunjak-Novakovic G., Burdick J. A., Rustgi A. K. (2021). Emerging technologies provide insights on cancer extracellular matrix biology and therapeutics. *iScience*.

[3] Winkler J., Abisoye-Ogunniyan A., Metcalf K. J., Werb Z. (2020). Concepts of extracellular matrix remodelling in tumour progression and metastasis. *Nature Communications*.

[4] Xing Y., Ruan G., Ni H. (2021). Tumor immune microenvironment and its related miRNAs in tumor progression. *Frontiers in Immunology*.

[5] Palumbo A., Meireles Da Costa N., Pontes B. (2020). Esophageal cancer development: crucial clues arising from the extracellular matrix. *Cells*.

[6] Huang X., Lan Y., Li E., Li J., Deng Q., Deng X. (2021). Diagnostic values of MMP-7, MMP-9, MMP-11, TIMP-1, TIMP-2, CEA, and CA19-9 in patients with colorectal cancer. *The Journal of International Medical Research*.

[7] Kim E. S., Nam S. M., Song H. K. (2021). CCL8 mediates crosstalk between endothelial colony forming cells and triple-negative breast cancer cells through IL-8, aggravating invasion and tumorigenicity. *Oncogene*.

[8] Zhao Y., Zheng X., Zheng Y. (2021). Extracellular matrix: emerging roles and potential therapeutic targets for breast cancer. *Frontiers in Oncology*.

[9] Schips T. G., Vanhoutte D., Vo A. (2019). Thrombospondin-3 augments injury-induced cardiomyopathy by intracellular integrin inhibition and sarcolemmal instability. *Nature Communications*.

[10] de Fraipont F., Nicholson A. C., Feige J. J., Van Meir E. G. (2001). Thrombospondins and tumor angiogenesis. *Trends in Molecular Medicine*.

[11] Liu X., Xu D., Liu Z. (2020). THBS1 facilitates colorectal liver metastasis through enhancing epithelial-mesenchymal transition. *Clinical and Translational Oncology*.

[12] Grunberg N., Pevsner-Fischer M., Goshen-Lago T. (2021). Cancer-associated fibroblasts promote aggressive gastric cancer phenotypes via heat shock factor 1-mediated secretion of extracellular vesicles. *Cancer Research*.

[13] Ao R., Guan L., Wang Y., Wang J. N. (2018). Silencing of COL1A2, COL6A3, and THBS2 inhibits gastric cancer cell proliferation, migration, and invasion while promoting apoptosis through the PI3k-Akt signaling pathway. *Journal of Cellular Biochemistry*.

[14] El Rayes T., Catena R., Lee S. (2015). Lung inflammation promotes metastasis through neutrophil protease-mediated degradation of Tsp-1. *Proceedings of the National Academy of Sciences*.

[15] Lu Y., Kong X. H., Zhong W. J., Hu M. H., Li C. J. (2021). Diagnostic, therapeutic, and prognostic value of the thrombospondin family in gastric cancer. *Frontiers in Molecular Bioscience*.

[16] Albo D., Shinohara T., Tuszynski G. P. (2002). Up-regulation of matrix metalloproteinase 9 by thrombospondin 1 in gastric cancer. *Journal of Surgical Research*.

[17] Liu J. F., Chen P. C., Chang T. M., Hou C. H. (2020). Thrombospondin-2 stimulates MMP-9 production and promotes osteosarcoma metastasis via the PLC, PKC, c-Src and NF-kappaB activation. *Journal of Cellular and Molecular Medicine*.

[18] Dalla-Torre C. A., Yoshimoto M., Lee C.-H. (2006). Effects of THBS3, SPARC and SPP1 expression on biological behavior and survival in patients with osteosarcoma. *BMC Cancer*.

[19] Kim M. S., Choi H. S., Wu M. (2020). Potential role of PDGFRbeta-associated THBS4 in colorectal cancer development. *Cancers (Basel)*.

[20] Shi Y., Sun L., Zhang R. (2021). Thrombospondin 4/integrin alpha2/HSF1 axis promotes proliferation and cancer stem-like traits of gallbladder cancer by enhancing reciprocal crosstalk between cancer-associated fibroblasts and tumor cells. *Journal of Experimental & Clinical Cancer Research*.

[21] Guo D., Zhang D., Ren M. (2020). THBS4 promotes HCC progression by regulating ITGB1 via FAK/PI3K/AKT pathway. *The FASEB Journal*.

[22] Zhang J., Wang H., Lv C. (2021). Cartilage oligomeric matrix protein affects the biological behavior of papillary thyroid carcinoma cells by activating the PI3K/AKT/Bcl-2 pathway. *Journal of Cancer*.

[23] Liu X., Cai S., Zhang C. (2018). Deacetylation of NAT10 by Sirt1 promotes the transition from rRNA biogenesis to autophagy upon energy stress. *Nucleic Acids Research*.

[24] Liu X., Su K., Sun X. (2021). Sec62 promotes stemness and chemoresistance of human colorectal cancer through activating Wnt/beta-catenin pathway. *Journal of Experimental & Clinical Cancer Research*.

[25] Tang Z., Li C., Kang B., Gao G., Li C., Zhang Z. (2017). GEPIA: a web server for cancer and normal gene expression profiling and interactive analyses. *Nucleic Acids Research*.

[26] Gao J., Aksoy B. A., Dogrusoz U. (2013). Integrative analysis of complex cancer genomics and clinical profiles using the cBioPortal. *Science Signaling*.

[27] Liu C. J., Hu F. F., Xia M. X., Han L., Zhang Q., Guo A. Y. (2018). GSCALite: a web server for gene set cancer analysis. *Bioinformatics*.

[28] Zhou K. R., Liu S., Sun W. J. (2017). ChIPBase v2.0: decoding transcriptional regulatory networks of non-coding RNAs and protein-coding genes from ChIP-seq data. *Nucleic Acids Research*.

[29] Sticht C., De La Torre C., Parveen A., Gretz N. (2018). miRWalk: an online resource for prediction of microRNA binding sites. *PLoS One*.

[30] Li J. H., Liu S., Zhou H., Qu L. H., Yang J. H. (2014). starBase v2.0: decoding miRNA-ceRNA, miRNA-ncRNA and protein-RNA interaction networks from large-scale CLIP-Seq data. *Nucleic Acids Research*.

[31] Vasaikar S. V., Straub P., Wang J., Zhang B. (2018). Linkedomics: analyzing multi-omics data within and across 32 cancer types. *Nucleic Acids Research*.

[32] Smirnov P., Kofia V., Maru A. (2018). PharmacoDB: an integrative database for mining in vitro anticancer drug screening studies. *Nucleic Acids Research*.

[33] Li T. W., Fan J. Y., Wang B. B. (2017). A web server for comprehensive analysis of tumor-infiltrating immune cells. *Cancer Research*.

[34] Szklarczyk D., Gable A. L., Lyon D. (2019). STRING v11: protein-protein association networks with increased coverage, supporting functional discovery in genome-wide experimental datasets. *Nucleic Acids Research*.

[35] Otasek D., Morris J. H., Bouças J., Pico A. R., Demchak B. (2019). Cytoscape automation: empowering workflow-based network analysis. *Genome Biology*.

[36] Zhang J., Ren P., Xu D. (2019). Human UTP14a promotes colorectal cancer progression by forming a positive regulation loop with c-Myc. *Cancer Letters*.

[37] Chockley P. J., Keshamouni V. G. (2016). Immunological consequences of epithelial-mesenchymal transition in tumor progression. *The Journal of Immunology*.

[38] Zhou J., Tang Z., Gao S., Li C., Feng Y., Zhou X. (2020). Tumor-associated macrophages: recent insights and therapies. *Frontiers in Oncology*.

[39] Horiguchi H., Yamagata S., Rong Qian Z., Kagawa S., Sakashita N. (2013). Thrombospondin-1 is highly expressed in desmoplastic components of invasive ductal carcinoma of the breast and associated with lymph node metastasis. *The Journal of Medical Investigation: JMI*.

[40] Xiao M., Zhang J., Chen W., Chen W. (2018). M1-like tumor-associated macrophages activated by exosome-transferred THBS1 promote malignant migration in oral squamous cell carcinoma. *Journal of Experimental & Clinical Cancer Research*.

[41] Wang P., Zeng Z., Lin C. (2020). Thrombospondin-1 as a potential therapeutic target: multiple roles in cancers. *Current Pharmaceutical Design*.

[42] Daubon T., Léon C., Clarke K. (2019). Deciphering the complex role of thrombospondin-1 in glioblastoma development. *Nature Communications*.

[43] Tian Q., Liu Y., Zhang Y. (2018). THBS2 is a biomarker for AJCC stages and a strong prognostic indicator in colorectal cancer. *Journal of BUON: Official Journal of the Balkan Union of Oncology*.

[44] Chang I. W., Li C. F., Lin V. C. H. (2016). Prognostic impact of thrombospodin-2 (THBS2) Overexpression on patients with urothelial carcinomas of upper urinary tracts and bladders. *Journal of Cancer*.

[45] Deng L. Y., Zeng X. F., Tang D., Deng W., Liu H. F., Xie Y. K. (2021). Expression and prognostic significance of thrombospondin gene family in gastric cancer. *Journal of Gastrointestinal Oncology*.

[46] Rahman M. T., Muppala S., Wu J. (2020). Effects of thrombospondin-4 on pro-inflammatory phenotype differentiation and apoptosis in macrophages. *Cell Death & Disease*.

[47] Förster S., Gretschel S., Jöns T., Yashiro M., Kemmner W. (2011). THBS4, a novel stromal molecule of diffuse-type gastric adenocarcinomas, identified by transcriptome-wide expression profiling. *Modern Pathology*.

[48] Chen X., Huang Y., Wang Y., Wu Q., Hong S., Huang Z. (2019). THBS4 predicts poor outcomes and promotes proliferation and metastasis in gastric cancer. *Journal of Physiology and Biochemistry*.

[49] Muppala S., Xiao R., Krukovets I. (2017). Thrombospondin-4 mediates TGF-beta-induced angiogenesis. *Oncogene*.

